# C-REACTIVE PROTEIN IN DIABETIC PATIENTS BEFORE GASTRIC BYPASS AS A POSSIBLE MARKER FOR POSTOPERATIVE COMPLICATION

**DOI:** 10.1590/S0102-6720201500S100005

**Published:** 2015-12

**Authors:** Daniel C. LINS, Josemberg M. CAMPOS, Patrícia S. de PAULA, Manoel GALVÃO-NETO, Eduardo PACHU, Ney CAVALCANTI, Álvaro A. B. FERRAZ

**Affiliations:** 1 Service of Endocrinology, Oswaldo Cruz Hospital, Faculty of Medical Sciences of Pernambuco; 2 Service of General Surgery/Post-graduation in Surgery, Federal University of Pernambuco, Recife, PE, Brazil

**Keywords:** Bariatric Surgery, Diabetes Mellitus, Type 2, Obesity, Roux-en-Y anastomosis, Gastric bypass, C-reactive protein, Postoperative complications

## Abstract

**Background:**

: Obesity and type 2 diabetes mellitus are associated to inflammatory state, which can be set off by the adipose tissue, once it is a metabolically active organ that can cause a chronic mild inflammatory state.

**Aim:**

: To evaluate the correlation between preoperative C-reactive protein and postoperative complications risk in obese patients (grades II and III) after Roux-en-Y gastric bypass, with and without type 2 diabetes mellitus.

***Methods*:**

Between 2008 and 2013 were analysed 209 patients (107 with diabetes), presenting body mass index >40 kg/m^2^or >35 kg/m^2^with comorbidities. During the postoperative period, two groups were evaluated: with and without complications. Preoperative ultra-sensitive C-reactive protein was measured by immunonephelometry method.

**Results:**

: Complications occurred in seven patients (pulmonary thromboembolism, fistula, two cases of suture leak, pancreatitis, evisceration and upper digestive hemorrhage). No statistical significance was found regarding lipid profile and C-reactive protein between patients with and without type 2 diabetes mellitus. When compared to each other, both groups (with and without complications) presented with statistical significance regarding C-reactive protein level (7,2 mg/dl vs 3,7 mg/dl, p=0,016) and had similar weight loss percentage after 3, 6 and 12 months follow-up.

**Conclusions:**

: Preoperative C-reactive protein serum level was higher in the group which presented complications after Roux-en-Y gastric bypass when compared to the group without postoperative complications.

## Abstract (pt)

**Racional:**

: Obesidade e diabete melito tipo 2 são associadas a estado inflamatório desencadeado pelo tecido adiposo, uma vez que é órgão metabolicamente ativo e que pode gerar estado de inflamação crônica leve.

**Objetivo:**

: Avaliar a correlação entre os níveis de proteína C reativa pré-operatória e o risco de complicações pós-operatórias em obesos graus II e III após bypass gástrico em Y-de-Roux, em um grupo com e outro sem diabete melito tipo 2.

**Métodos:**

: Entre 2008 e 2013 foram analisados 209 pacientes (107 diabéticos) com índice de massa corpórea acima de 40 kg/m^2^ ou maior que 35 kg/m^2^com comorbidades. No pós-operatório foi avaliado um grupo com e outro sem complicações. A proteína C reativa ultrassensível pré-operatória foi mensurada pela técnica de imunonefelometria.

**Resultados:**

: Complicações ocorreram em sete pacientes (tromboembolismo pulmonar, fístula, dois casos de vazamento de sutura, pancreatite, evisceração e hemorragia digestiva alta). Não houve diferença estatística significativa em relação ao perfil lipídico e níveis séricos de proteína C reativa entre os grupos com e sem diabete. Quando comparados, os dois grupos (com e sem complicações) apresentaram significância estatística entre os níveis de proteína C reativa (7,2 mg/dl vs 3,7 mg/dl, p=0,016), sendo semelhantes em relação à percentagem de perda de peso após seguimento de 3, 6 e 12 meses.

**Conclusões:**

: O nível sérico de proteína C reativa pré-operatória foi mais elevado no grupo com complicações após bypass gástrico em Y-de-Roux do que no grupo sem complicações.

## INTRODUCTION

The prevalence of overweight and obesity is growing throughout the world, increasing the frequency of comorbidities such as type 2 diabetes mellitus (DM2). Obesity and DM2 are conditions frequently associated with a chronic inflammatory state^28^. This association may be explained by the increase in circulating levels of various inflammatory markers, such as pro-inflammatory cytokines and proteins of the acute phase, such as interleukin 6 (IL-6), tumor necrosis factor α and C-reactive protein (CPR). The latter plays an important role in the response to systemic inflammation, increasing the plasma concentration during inflammatory processes^6^^,^ 24.

Bariatric surgery is a safe process with a mortality rate of less than 0.3%^5^. Early complications may occur in up to 13% of patients^23^. There are, however, no markers to safely determine the risk of postoperative complications. There are no studies in the literature that assess the relation between levels of pre-operative CPR and the risk of complications after Roux-en-Y gastric bypass (RYGB).

Some studies that involve different surgical specialties report higher serum levels of CPR with infectious postoperative complications^8^^,^ 17. The increase in pre-operative CPR was considered a risk factor for infection and an increase in hospital mortality for heart surgery, as well as increasing the risk of suture leakage and other infectious complications after elective colorectal surgery^8^^,^ 29. 

There is evidence of an association between the accumulation of adipose tissue and an increase in levels of CPR and adipose tissue is known to be a metabolically active organ that may cause a low-intensity chronic inflammatory state^11^^,^ 20 ^,^ 22 ^,^ 25.

The effect of weight loss after bariatric surgery on levels of inflammatory cytokines and markers of inflammation has been demonstrated^13^^,^ 30. After the surgical procedure, there is a reduction in CPR serum levels (65%, on average), according to weight loss^3^. However, there is no studies that assess the relation between heightened levels of CPR prior to RYGB and the occurrence of postoperative complications.

The aim of the present study was to assess the correlation between pre-operative levels of CPR and the risk of complications in individuals with grade II and grade III obesity after RYGB in one group with and one without DM2.

## METHODS

Two hundred and nine patients (65 men and 144 women) who had undergone RYGB at the General Surgery Service of the Federal University of Pernambuco's Hospital das Clínicas, Recife, PE, Brazil between 2008 and 2013 were analyzed retrospectively. The mean age was 40.2 years and the mean body mass index (BMI) 41.5 kg/m^2^. Type 2 diabetes mellitus was present in 107 patients, as diagnosed according to the criteria of the American Diabetes Association. The mean time for diagnosis of DM2 was 4.4 years. Bariatric surgery was recommended when BMI was greater than 40 kg/m^2^ or 35 kg/m^2^ with comorbidities.

The ultrasensitive CPR was measured preoperatively using the immunonephelometry technique. For evaluation of inflammatory/infectious processes the reference value used was greater than 5 mg/dl. The reference values used for evaluation of cardiovascular risk were as follows: low risk: <1.00 mg/l; moderate risk: 1.00 - 3.00 mg/dl; high risk: >3.00 mg/dl.

Serum biochemical tests were carried out by collecting blood samples (20 ml) from a peripheral vein by a single puncture in the morning on the day before the operation, after a period of rest and minimum fasting of 8 h. Laboratory tests included fasting glycemia, total cholesterol and fractions, and triglycerides.

Total cholesterol and fractions were measured in serum using an Analisa diagnostic kit, based principally on the colorimetric enzyme method. The desirable reference level for cholesterol was <200 mg/dl and for triglycerides <150 mg/dl.

All patients underwent laparoscopic RYGB with the creation of a gastric pouch with an approximate volume of 50 ml, a biliopancreatic diversion 100 cm in length and a 150 cm alimentary diversion. Intraoperative glucose was monitored using capillary glycemia and corrected with regular subcutaneous insulin, when necessary, to keep glycemia between 80 and 140 mg/dl.

**FIGURE 1:**
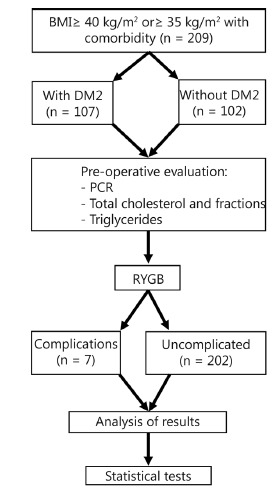
- Flow Chart

The patients were divided into two groups: with and without postoperative complications (up to 30 days). Outpatient follow-up began 15 days after the RYGB, with further consultations after 1, 2, 6 and 12 months, followed by at least one consultation per year (Figure 1).

The level of significance adopted for statistical tests was 5%. Data analysis was carried out using inferential statistical techniques and Student's t and the Mann-Whitney tests.

## RESULTS

The patients (with and without DM2) had a similar preoperative lipid and inflammatory profiles (Table 1). 

**FIGURE 2:**
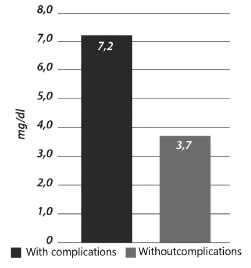
- Preoperative CRP vs surgical complications

After the RYGB, the data from the groups with and without complications were analyzed. Complications occurred in seven patients: pulmonary thromboembolism, fistula, suture leakage (n=2), pancreatitis, evisceration and upper digestive tract hemorrhage. The group with complications had a significantly higher level of CRP than the one without (p=0.016) (Figure 2).

There was no difference in weight loss after 3, 6 and 12 months between the two groups. The occurrence of surgical complications did not influence weight loss after follow-up. The mean weight loss after one year of follow-up was 33.42 kg (±6.97) and 33.79 kg (±5.80) in patients with and without complications, respectively (Tables 2 and 3).

**TABLE 1:** - Comparison of diabetic and non-diabetic groups

**Variables**	**Complications**	**p**	
	**Yes**	**No**	
·BMI	41,13 kg/m^2^ (± 4,84)	41,22 kg/m^2^ (± 4,72)	p^1^=0,786
· Weight loss after 3 months	18,73 kg (± 2,29)	17,78 kg (± 3,20)	p^1^=0,340
· Weight loss after 6 months	25,67 kg (± 2,21)	25,23 kg (±4,93)	p^1^ =0,777
· Weight loss after 1 year	33,42 kg (± 6,97)	33,79 kg (±5,80)	p^1^ =0,758

(1)=Mann-Whitney Test

**TABLE 2:** - Comparison of weight loss in groups with and without complications

**Variables**	**Diabetes**	**p**	
	**Yes**	**No**	
· Total cholesterol	208.09 mg/dl (± 42.52)	200.88 mg/dl (± 37.20)	p^1^ = 0.249
· Triglycerides	184.75 mg/dl (± 94.84)	160.60 mg/dl (± 100.18)	p^1^ = 0.135
· CPR	3.86 mg/dl (± 0.64)	3.65 mg/dl (± 0.42)	p^1^ = 0.640

**TABLE 3:** - Variables for group with complications

**n**	**Age**	**Gender**	**BMI pre-op.**	**CRP pre-op.**	**HbA1c pre-op.**	**Insulin pre-op.**	**Cholest. Total**	**Triglyc.**	**Weight loss**	**Weight loss**	**Weight loss**
			**(Kg/m** ^2^ **)**	**(mg/dL)**	**(mg/dL)**	**(mg/dL)**	**(mg/dL)**	**(mg/dL)**	**(kg) - 3 months**	**(kg) - 6 months**	**(kg) - 1 year**
1	34	M	45,2	8,5	6,3	23,9	227	182	18,6	26,2	36,5
2	46	F	42,5	4,5	6	19	259	135	16,5	24,8	32,2
3	44	M	35	7,8	3,1	16	314	628	23,9	27	30,4
4	64	F	37	4,4	6,2	11,5	247	180	18	26,5	24,4
5	32	F	40,5	10,3	5,3	38	179	185	21	23,2	26,5
6	58	M	49,7	7,6	5,8	14	304	700	20,9	25,1	30,7
7	41	M	41,3	3,9	5,2	5,2	237	215	15,8	18,4	18,1

n =patient; BMI=body mass index; pre-op= preoperative; CRP=C-reactive protein; Cholest. Total=total cholesterol; Triglyc.=triglycerides.

## DISCUSSION

The inflammatory profile as a marker for postoperative complications has been studied for some surgical specialties by measuring CRP^2^^,^ 8 ^,^ 17 ^,^ 29. However, there are few reports of the relation between serum levels of CRP and complications after RYGB^21^^,^ 27.

Obese patients may exhibit higher levels of CRP owing to the increase in production of interleukin-6 and tumor necrosis factor in adipocytes, regulating the production of CRP in the liver and inducing a low level chronic inflammatory state^19^^,^ 22 ^,^ 24. Publications have shown that patients with DM2 have higher levels of CRP and this may also indicate that obese patients with high levels of this protein are at greater risk of developing DM2^10^.

Hyperglycemia and DM2 have been associated with higher postoperative morbidity^30^. Some authors thus argue that most hospitalized diabetic patients should receive insulin therapy, according to the severity of the DM2, to reduce the risk of complications^25^. 

In the present study there was no difference in plasma levels of CRP, triglycerides and total cholesterol between diabetics and non-diabetics. This similarity may be explained by the fact that the patients without diabetes were severely obese and thus exhibited strong inflammatory activity, heightened cardiovascular risk and a greater likelihood of progression to DM2^4^^,^ 9. Furthermore, the patients with DM2 included in the present study had, on average, only had the disease for a short time. The small sample size should also be considered. 

The identification of factors associated with higher risk of surgical complications is important for adequate selection of patients prior to surgery. The classification of these according to degrees of surgical risk enables improved quality of surgical treatment and outcomes^14^^,^ 15. 

RYGB has a low complications rate compared to other surgical procedures. The incidence of early postoperative complications, such as fistula, varies, on average, from 0.4 to 5.2% in most studies^12^. However, these complications may be difficult to treat, requiring early intervention and exhibiting high levels of morbidity^7^. Identification of a serum risk factor marker for postoperative complications, such as CPR, may, therefore, be a predictor for severe complications after RYGB, reducing the risks of the procedure.

The present study identified a preoperative CRP of over 3 mg/dl as presenting a higher risk of postoperative complications. It has been shown that preoperative measurement of CRP or the CRP curve is important for stratifying the risk of early surgical complications^1^^,^ 17 ^,^ 26. 

In vascular lower-limb by-pass surgery, patients with CRP greater than 5 mg/dl immediately prior to surgery had a higher risk of postoperative vascular complications. Follow-up showed that 60% of the patients (21/35) had complications compared to 32% (18/56) for the group with CRP of less than 5 mg/dl (p=0.004)^17^.

A study published in 2012 conducted a retrospective analysis of patients undergoing laparoscopic RYGB. Of these, 4.1% (n=17) developed a fistula after an average of five days. Higher levels of CRP two days after RYGB proved to be of great diagnostic value for predicting postoperative complications especially intestinal leakage^27^. 

In general, older patients and those with comorbidities, principally diabetes and hypertension, have a greater risk of developing a fistula and a higher risk of death. This group of patients should thus be avoided at the beginning of the learning curve in order to prevent postoperative complications. Preoperative serum levels of CRP may be a predictor of the occurrence of complications, such as fistulas. 

Despite the relation between heightened levels of CRP and the occurrence of postoperative complications having been suggested by various studies, some appear to disagree with this hypothesis. In a meta-analysis carried out by Padayache, in 2009, heightened levels of CRP were not found to be related to a higher incidence of morbidity and/or mortality up to 30 days after surgery^18^.

The possibility of serum markers predictive of complications other than CRP has also been investigated. In a non-randomized prospective cohort study published in 2011, levels of procalcitonin were examined as a possible prognostic parameter for infectious complications in patients with acute spinal cord lesions with and without postoperative infection. The patients who experienced complications (7.7%) had significantly higher levels of procalcitonin and CRP compared to those without complications, with procalcitonin being more sensitive than CRP^16^.

The inflammatory marker CRP may have a role to play in the preoperative routine for grade II or III obesity patients who are candidates for bariatric surgery, as a marker for the risk of immediate postoperative complications or be used to make up part of the score for stratification of surgical risk. High levels of CRP may indicate the need to delay or suspend surgery until levels of this protein have been normalized by weight loss and better metabolic control, as a way of avoiding a higher occurrence of immediate postoperative complications.

## CONCLUSION

Preoperative serum levels of C reactive protein were higher in the group with complications after Roux-en-Y gastric by-pass surgery than in the one without complications.
